# Transfer-workflow optimization in a regional chest pain center network reduces door-in–door-out time in ST-segment elevation myocardial infarction: An interrupted time-series study with spectral validation

**DOI:** 10.1016/j.ijcha.2026.102020

**Published:** 2026-09-25

**Authors:** Xiaolei Ma, Xianjun Wu, Liang Wen, Ruifeng Wang, Jian Cao, Tong Zhang, Kun Bai, Zhongshang Guo, Yi Xie

**Affiliations:** a Coronary Care Unit (CCU), Hanzhong Central Hospital, Hanzhong 723000, China; b Department of Osteoarticular Surgery, Hanzhong Central Hospital, Hanzhong 723000, China

**Keywords:** ST-segment elevation myocardial infarction, Chest pain center, Inter-hospital transfer, Door-in–door-out, Interrupted time series, Workflow optimization

## Abstract

**Background:**

Inter-hospital transfer delays remain a major barrier to timely primary percutaneous coronary intervention (PCI) for patients with ST-segment elevation myocardial infarction (STEMI). We evaluated a structured transfer-workflow optimization program in a regional hub-and-spoke chest pain center network.

**Methods:**

This retrospective interrupted time series study included 785 transferred STEMI patients recorded in the National Chest Pain Center Reporting Platform within the Hanzhong Central Hospital regional network, China, from November 2019 to September 2025. A hub-led workflow intervention began in February 2022 and targeted reception, triage, STEMI recognition, internal pathways, and earlier hub notification at spoke hospitals. The primary endpoint was monthly mean door-in–door-out (DIDO) time; secondary endpoints were first medical contact–to–wire crossing (FMC2W) and in-hospital outcomes. Segmented regression was complemented by autocorrelation diagnostics, sensitivity models, spectral analyses, and negative-control analyses.

**Results:**

The intervention was associated with an immediate DIDO reduction of 49.7 min (*P* = 0.009), with consistent autocorrelation-robust estimates. Shorter DIDO was associated with shorter FMC2W, whereas non-targeted downstream intervals remained stable. Spectral analyses supported reduced high-frequency variability. Crude rates of composite complications and new-onset in-hospital heart failure were lower after intervention, but model-based effects at 12 and 24 months were not significant. These were fitted-model estimates, not patient-level follow-up; clinical endpoints were exploratory.

**Conclusions:**

Structured transfer-workflow optimization was associated with reduced DIDO time and shorter FMC2W. Clinical outcome findings were exploratory and did not establish long-term benefit.

## 1 Introduction

Acute ST-segment elevation myocardial infarction (STEMI) remains a major contributor to cardiovascular mortality worldwide, and its management continues to test the limits of health-system performance even in the era of mature reperfusion strategies [1]. The clinical maxim “time is muscle” is not rhetorical: the benefit of percutaneous coronary intervention (PCI) is tightly coupled to the rapidity with which coronary flow is restored [2], [3]. Yet, while procedural success rates have steadily improved, the logistics of delivering eligible patients to a catheterization laboratory have not advanced at the same pace—an imbalance that becomes particularly apparent when first presentation occurs at non–PCI-capable facilities [4], [5].

For patients diagnosed with STEMI at non–PCI-capable hospitals (spoke centers), contemporary guidelines call for expedited inter-hospital transfer to a PCI-capable hub, with door-in–door-out (DIDO) time ideally kept within 30 min [4]. Within many regions, this expectation has been operationalized through hub-and-spoke chest pain center networks intended to standardize triage, accelerate decision-making, and shorten the referral pathway [4]. Even so, delays at the referring hospital remain a persistent bottleneck: prolonged DIDO time has been associated with longer total ischemic time and higher rates of major adverse cardiovascular events (MACE) [5], [6]. Accordingly, a wide range of quality-improvement approaches—spanning protocol checklists and telemedicine-supported activation—has been deployed to reduce system delay, with variable success across settings [7], [8], [9].

Despite sustained efforts, real-world performance frequently falls short of the recommended target, and registry data commonly report median DIDO times well above 30 min [6], [7]. What is less clear—and arguably more consequential for implementation science—is whether a discrete, structured workflow-training intervention can produce durable, intervention-specific improvement once background secular trends, seasonality, and autocorrelation are appropriately accounted for [10], [11]. Much of the existing evidence relies on pre–post comparisons that are intrinsically vulnerable to time-related confounding, and relatively few evaluations have separated the effect of targeted training from broader system maturation or corroborated temporal signals using complementary analytic approaches such as frequency-domain (spectral) methods [10], [11], [12]. Consequently, uncertainty persists regarding whether transfer-latency reductions achieved through training and process redesign are statistically stable over time and achievable without compromising downstream safety [10], [11], [12], [13].

To address these gaps, the impact of a structured inter-hospital transfer-workflow optimization program implemented within a regional chest pain center network was evaluated using an interrupted time series (ITS) design. Immediate and longitudinal changes were quantified in key efficiency metrics, including DIDO time and first medical contact–to–wire crossing (FMC2W), used as a system-level proxy for total ischemic time. Beyond segmented regression, prespecified validation strategies were applied to interrogate the specificity and robustness of the estimated intervention signal, incorporating frequency-domain (spectral) analysis and negative control outcomes. It was hypothesized that training-centered workflow optimization at spoke centers would yield a prompt and sustained reduction in DIDO time, be associated with downstream shortening of FMC2W, and provide exploratory evidence on selected in-hospital outcomes.

Classical ITS analysis is well suited to estimate whether an intervention is associated with an abrupt level change or a change in post-intervention trend. However, ITS alone provides limited information on whether a process becomes more stable over time or whether observed changes primarily reflect random high-frequency fluctuation or recurring cyclic variability. Frequency-domain spectral analysis can therefore complement ITS by characterizing how variance is distributed across temporal frequencies, providing supportive evidence for changes in process stability after workflow optimization. Published evaluations of STEMI transfer and workflow optimization have commonly used before–after comparisons or conventional time-trend analyses [7], [8], [9], [14]. Relatively few studies have combined an extended ITS framework with frequency-domain validation, negative-control interval analyses, and a DIDO-to-FMC2W pathway assessment. Accordingly, the present study was designed to evaluate not only whether DIDO decreased after a hub-led workflow intervention, but also whether the signal was temporally stable, specific to the targeted transfer process, and associated with downstream shortening of FMC2W.

## 2 Methods

### 2.1 Study design and setting

We conducted a retrospective interrupted time series (ITS) study within a regional hub-and-spoke chest pain center network in Hanzhong, Shaanxi Province, China. Hanzhong Central Hospital is the PCI-capable regional hub and tertiary referral center for this network. The affiliated spoke sites are chest pain units without on-site catheterization laboratory or primary PCI capability; therefore, STEMI patients initially presenting to these non-PCI-capable units require inter-hospital transfer to the hub for coronary angiography and PCI. Patient records were obtained from the National Chest Pain Center Reporting Platform and aggregated into monthly time series from November 2019 to September 2025, enabling estimation of immediate level and post-intervention trend changes. The study protocol was reviewed and approved by the Institutional Review Board (IRB) of Hanzhong Central Hospital (Approval No. 2025-Lun-Wei-Shen-128). Due to the retrospective nature of the study and the use of de-identified data, the requirement for informed consent was waived by the IRB. All procedures were conducted in accordance with the Declaration of Helsinki.

The network operates as a semi-centralized system. Standardized protocols, training, quality feedback, and hub-notification pathways are coordinated by the PCI-capable hub, whereas initial triage, STEMI recognition, first ECG/laboratory testing, and transfer initiation are performed locally within each spoke unit. The intervention therefore targeted the spoke-side processes that determine DIDO time.

### 2.2 Study population

Consecutive patients with ST-segment elevation myocardial infarction (STEMI) who initially presented at affiliated chest pain units and were transferred to the hub center were included. Patients with missing key timestamp data required to derive transfer intervals were excluded from the corresponding time-interval analysis.

De-identified structured registry records were obtained from the National Chest Pain Center Reporting Platform for all eligible transferred STEMI cases within the predefined study window. Data extraction was based on predefined platform fields and automated structured-data processing. All cases from November 2019 to September 2025 in the regional network were included without subjective case selection. Field mapping, derived time intervals, and logical consistency were checked before analysis by the study team. Formal blinding to the study hypothesis was not feasible because of the retrospective registry-based design; however, use of standardized platform fields minimized subjective ascertainment.

### 2.3 Intervention

The intervention was embedded within the regional chest pain center framework and consisted of a hub-led, spoke-side optimization of the inter-hospital transfer workflow for patients presenting to non-PCI-capable chest pain units. Before the intervention, spoke units varied in early STEMI recognition, in-hospital routing, hub notification, and transfer activation; some patients experienced diagnostic delay or non-standardized referral pathways before transfer. The hub team therefore provided hands-on training and protocol optimization to each affiliated chest pain unit. The intervention targeted four operational domains: (1) dedicated reception and triage roles for chest pain patients, supported by enhanced in-hospital signage; (2) staff training for early STEMI recognition and standardized ECG interpretation; (3) internal workflow optimization for rapid ECG acquisition, laboratory testing, consultation, and transfer preparation; and (4) accelerated referral mechanisms, including earlier hub notification, predefined transfer contacts, and standardized transfer-bypass procedures. The intervention was formally implemented in February 2022, which served as the predefined interruption point for the ITS analysis. Hub-side catheterization-laboratory workflows at Hanzhong Central Hospital were not structurally changed and were not the target of the intervention. Detailed components of the transfer-workflow optimization intervention are summarized in Supplementary Table S1.

### 2.4 Outcomes and measures

#### 2.4.1 Transfer time metrics

Transfer metrics were derived from standardized registry timestamps. DIDO was calculated as departure time from the spoke hospital minus arrival time at the spoke hospital. D2D was calculated as arrival time at the hub hospital minus departure time from the spoke hospital. D2W was calculated as wire-crossing time minus arrival time at the hub hospital. FMC2W was calculated as wire-crossing time minus first medical contact time. Each interval was calculated directly from two corresponding platform timestamp fields, and no subjective selection among multiple competing timestamp candidates was required. Detailed operational definitions are provided in Supplementary Table S2.

#### 2.4.2 Clinical outcomes

Clinical outcomes were assessed during the index hospitalization, from admission until discharge or in-hospital death. The registry-defined composite complications endpoint was coded as present when at least one of the following occurred: heart failure, bleeding, malignant arrhythmia, infection, respiratory failure, mechanical complication, ischemic stroke, renal failure, death, shock, or recurrent myocardial infarction. Multiple complications in the same patient were counted once for the composite endpoint. Heart failure and death, both components of the composite endpoint, were also analyzed separately as clinically relevant component outcomes.

### 2.5 Statistical analysis

#### 2.5.1 Data aggregation, outlier handling, and interrupted time series modeling

Monthly means were used as the primary aggregation metric for time-interval outcomes. To minimize the influence of extreme transfer delays attributable to atypical clinical pathways, individual observations with DIDO values above the period-specific 95th percentile were excluded before calculating monthly means; details of the trimming strategy are provided in Supplementary Table S3. The segmented regression model included a continuous time variable, an intervention indicator, and a post-intervention time variable to estimate the baseline trend, immediate level change, and change in post-intervention slope. The ITS model was specified as:

$$Y_{t}=\beta_{0}+\beta_{1}\cdot \mathrm{tim}e_{t}+\beta_{2}\cdot \mathrm{interventio}n_{t}+\beta_{3}\cdot \mathrm{time}\_\mathrm{afte}r_{t}+\varepsilon_{t}$$

where $Y_{t}$ denotes the monthly mean outcome at month t; $\mathrm{tim}e_{t}$ is a continuous time variable indicating months since the start of the observation period; $\mathrm{interventio}n_{t}$ is a binary indicator coded 0 for the pre-intervention period and 1 for the post-intervention period (February 2022 onward); and $\mathrm{time}\_\mathrm{afte}r_{t}$ equals 0 before the intervention and counts months since the intervention thereafter. In this parameterization, $\beta_{0}$ is the baseline level at t=0, $\beta_{1}$ represents the pre-intervention trend (slope), $\beta_{2}$ estimates the immediate level change at the interruption point, and $\beta_{3}$ captures the change in slope after the intervention relative to the pre-intervention trend; $\varepsilon_{t}$ is the error term.

#### 2.5.2 Model specification, seasonality, and inference

Model diagnostics were expanded to evaluate residual autocorrelation and the robustness of inference. Residual autocorrelation was assessed using residual ACF/PACF plots, the Durbin–Watson test, and the Ljung–Box test. Because these diagnostics suggested residual serial correlation, we additionally estimated Newey–West robust standard errors and a generalized least-squares model with an AR(1) correlation structure. Seasonal adjustment and alternative time-trend specifications were evaluated as sensitivity analyses. Graphical diagnostics for the primary DIDO interrupted time series model are shown in **Supplementary Fig. S6**, and formal diagnostics and robust ITS estimates are reported in **Supplementary Table S4**.

#### 2.5.3 Segmented logistic regression for binary clinical outcomes

For composite complications, new-onset in-hospital heart failure, and in-hospital mortality, patient-level segmented logistic regression models included terms for the baseline time trend, immediate post-intervention level change (β2), and post-intervention slope change (β3). Effect estimates are reported as odds ratios with 95% confidence intervals for event occurrence. The model-based combined effects at 12 and 24 months after the intervention were calculated as exp.(β2 + 12β3) and exp.(β2 + 24β3), respectively. These quantities describe the estimated intervention effect at specified points in post-intervention calendar time and are not patient-level 12- or 24-month follow-up outcomes. Full segmented logistic regression estimates are reported in Supplementary Table S5. Because mortality was a low-frequency endpoint, a Firth penalized logistic regression model was additionally used as a small-sample sensitivity analysis.

#### 2.5.4 Spectral and time-series decomposition analysis

Frequency-domain analyses, including power spectral density estimation, were used as complementary exploratory analyses to characterize dominant periodic components and assess whether high-frequency variability in DIDO time was attenuated after the intervention. Detailed spectral components and robustness analyses are reported in Supplementary Tables S6A and S6B.

#### 2.5.5 Mechanistic and specificity analyses

We evaluated the association between monthly mean DIDO and FMC2W to examine whether improvement in spoke-side transfer efficiency was accompanied by shorter FMC2W, used as a system-level proxy for total ischemic time. Pearson correlation coefficients with 95% confidence intervals and Spearman rank correlations were calculated overall and separately for the pre- and post-intervention periods. A DIDO-by-period interaction term was used to assess whether the DIDO–FMC2W relationship differed between periods. Specificity was assessed using DIDO versus D2D as a negative-control association, because D2D was not the primary target of the spoke-side intervention. The DIDO–FMC2W association, DIDO-by-period interaction, negative-control analysis, and mortality sensitivity analysis are summarized in Supplementary **Fig. S8** and **Supplementary Table S7**.

#### 2.5.6 Statistical considerations

All analyses were performed using R version 4.3.3. Time-series models, robust standard errors, generalized least-squares models, Firth logistic regression, and graphics were implemented using base R and relevant packages including lmtest, sandwich, nlme, logistf, dplyr, ggplot2, and related tidyverse utilities. A two-sided *P* value <0.05 was considered statistically significant.

## 3 Results

### 3.1 Study population and baseline characteristics

Among 827 eligible registry records, 42 observations with period-specific extreme DIDO values were excluded according to the prespecified 95th-percentile trimming strategy, yielding 785 patients for the primary analysis over 71 consecutive months. Monthly case volume fluctuated between 3 and 27 patients (Supplementary Fig. S1). Monthly means after outlier exclusion were used as the primary aggregation metric to reduce estimator variance in months with limited samples. Baseline demographic and clinical characteristics before and after the intervention are summarized in Supplementary Table S8, and included-versus-excluded patients are compared in Supplementary Table S9. Except for the markedly longer transfer intervals expected from the exclusion rule, baseline characteristics were broadly similar.

### 3.2 Primary effect of the intervention on transfer efficiency

#### 3.2.1 Interrupted time series results

Fig. 1 illustrates the temporal impact of the intervention on DIDO time. Immediately following implementation in February 2022, a marked reduction in DIDO time was observed (Fig. 1A). In the ITS model, the intervention was associated with an immediate level change of −49.7 min (*P* = 0.009), while pre-intervention trends and post-intervention slope changes were not statistically significant (Fig. 1A–B**, Supplementary Table S10**). Distributional analyses showed a leftward shift in DIDO times post-intervention, with mean values decreasing from 155 to 94 min and medians from 104 to 70 min (Fig. 1B). Sensitivity analyses across alternative model specifications and outlier-handling strategies supported the robustness of these findings (**Supplementary Fig. S2**). Autocorrelation diagnostics suggested residual serial correlation in the primary OLS segmented regression (Durbin–Watson statistic 1.685, *P* = 0.043; Ljung–Box statistic 26.454, *P* = 0.009). The estimated immediate post-intervention DIDO reduction remained consistent across robust model specifications: OLS-HC1 estimate −49.57 min (95% CI −85.72 to −13.41; *P* = 0.009), Newey–West estimate −49.57 min (95% CI −78.63 to −20.51; *P* = 0.001), and GLS-AR(1) estimate −49.33 min (95% CI −79.90 to −18.77; *P* = 0.002).

**Fig. 1:**
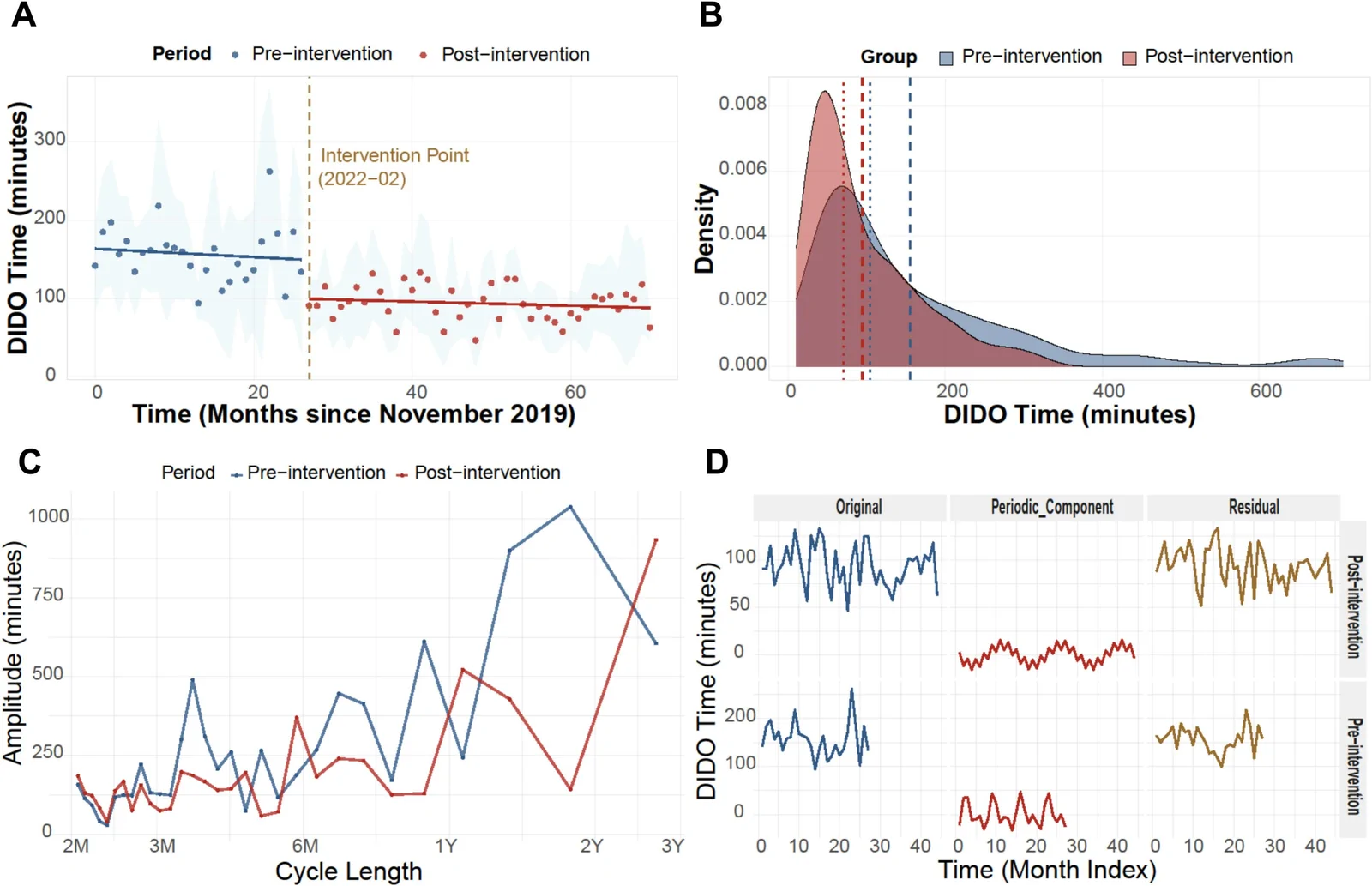
**Impact of the primary intervention on transfer efficiency, temporal dynamics, and spectral characteristics of DIDO time in chest pain units. (A) Monthly Trend of DIDO Time in Chest Pain Units (From Nov 2019). (B) Comparison of DIDO Time Distribution Before and After Intervention. (C) Spectral Analysis of DIDO Time Series. (D) Time Series Decomposition.** CI = confidence interval, DIDO = door-in–door-out, ITS = interrupted time series.

#### 3.2.2 Spectral validation of time-series signal

Frequency-domain analyses provided complementary support for the ITS findings. Power spectral density analysis suggested attenuated high-frequency variability and a reduced contribution of dominant periodic components after the intervention (Fig. 1C), consistent with improved temporal stability of the transfer process. Seasonal–trend decomposition demonstrated that the DIDO reduction was primarily a structural level change rather than seasonal variation (Fig. 1D). Detailed characterization of periodic components is provided in **Supplementary Fig. S3**. Sensitivity analyses incorporating seasonal adjustment yielded consistent intervention effect estimates (**Supplementary Fig. S2**).

### 3.3 Mechanistic pathway and specificity of the intervention

Mechanistic analyses showed a strong association between monthly mean DIDO and FMC2W (Fig. 2A–B, **Supplementary Fig. S8**, and **Supplementary Table S7A**). Specifically, monthly mean DIDO was strongly correlated with monthly mean FMC2W overall (Pearson *r* = 0.921, 95% CI 0.876–0.950; *P* < 0.001). This association remained directionally consistent in both the pre-intervention period (*r* = 0.957, 95% CI 0.907–0.980; *P* < 0.001) and the post-intervention period (*r* = 0.755, 95% CI 0.591–0.859; *P* < 0.001). The DIDO-by-period interaction was not statistically significant (interaction estimate −0.096, 95% CI −0.374 to 0.182; *P* = 0.502; **Supplementary Table S7B**), suggesting no clear evidence that the DIDO–FMC2W relationship differed after the intervention. Pathway-oriented association analyses were consistent with DIDO being the dominant contributor to FMC2W variation (β = 1.015, *P* < 0.001), but this should be interpreted as an association rather than formal causal mediation. Negative-control analyses showed no meaningful association between monthly mean DIDO and D2D time (Pearson *r* = −0.061, 95% CI −0.290 to 0.175; *P* = 0.614; Fig. 2C and **Supplementary Table S7C**), supporting the specificity of the observed DIDO–FMC2W relationship to the targeted transfer workflow rather than to non-targeted transport intervals. Quarterly aggregation further illustrated sustained declines in DIDO and FMC2W, while non-targeted intervals (D2D and D2W) remained stable (Fig. 2D).

**Fig. 2:**
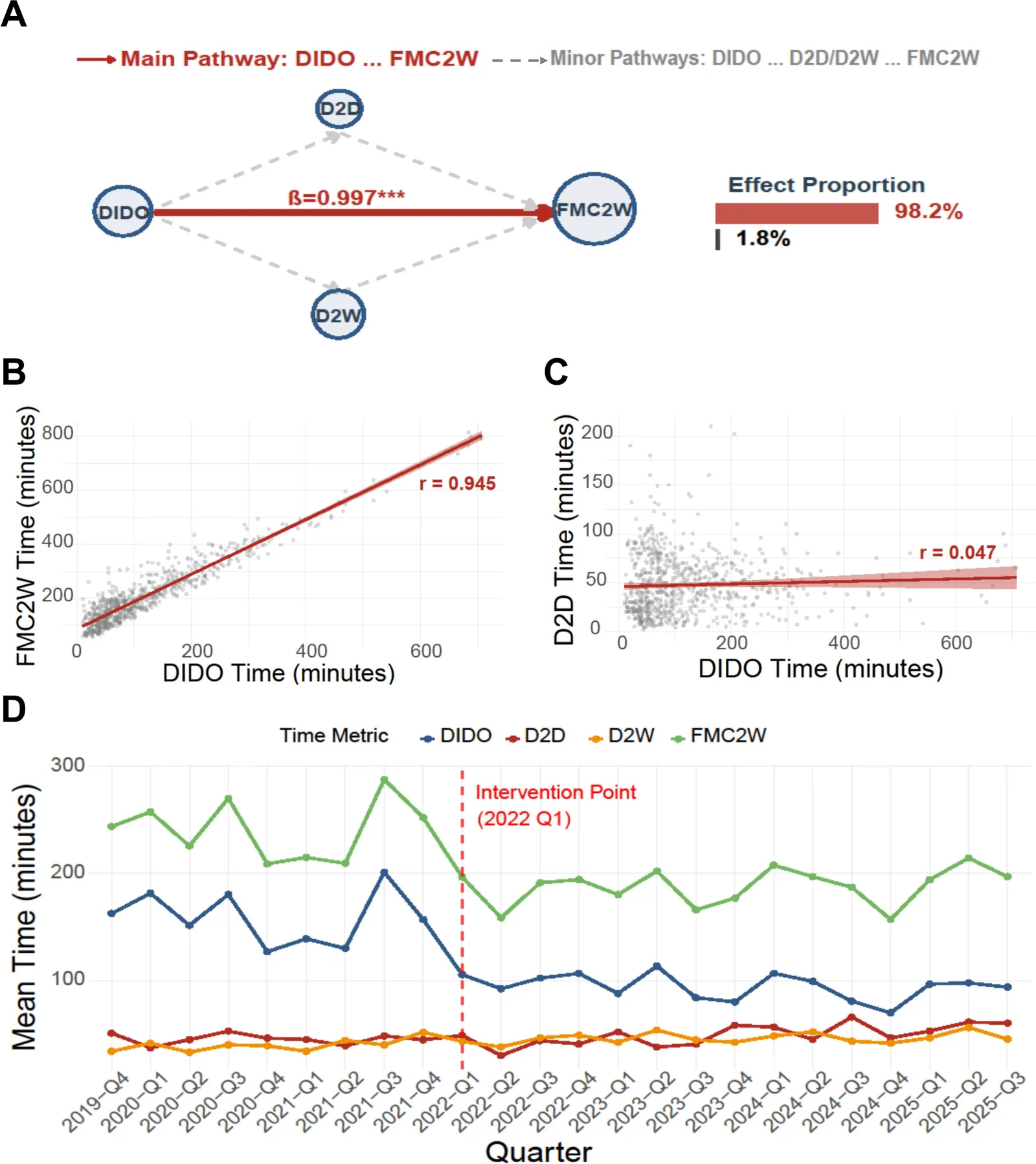
**Mechanistic pathway and specificity analyses. (A) Mechanism of DIDO on FMC2W: Direct Effect Dominance. (B) Overall Relationship between DIDO and FMC2W. (C) Overall Relationship between DIDO and D2D. (D) Quarterly Trends of Key Time Metrics.** D2D = door-out–to–door, D2W = door-to-wire crossing time, DIDO = door-in–door-out, FMC2W = first medical contact–to–wire crossing.

### 3.4 Clinical outcomes and resource utilization

Clinical outcomes are summarized in Table 1 and visualized in Fig. 3; quarterly event rates are shown in Supplementary Fig. S7 and Supplementary Table S11. In crude pre/post comparisons, composite complications decreased from 19.05% to 13.32% (risk difference, −5.73%; 95% CI, −10.92 to −0.54; *P* = 0.029), and new-onset in-hospital heart failure decreased from 12.61% to 7.48% (risk difference, −5.13%; 95% CI, −9.38 to −0.88; *P* = 0.016). In-hospital mortality did not differ significantly between periods (2.24% vs 3.50%; risk difference, 1.26%; 95% CI, −1.06 to 3.59; *P* = 0.296). These crude reductions were not consistently supported by the full segmented logistic models. Although the immediate level term for new-onset in-hospital heart failure was statistically significant, the model-based combined effects at 12 and 24 months after the intervention were not statistically significant for composite complications, new-onset in-hospital heart failure, or mortality. These estimates describe modeled changes in the odds of an in-hospital event at specified calendar times after the intervention, not outcomes after 12 or 24 months of patient follow-up. Clinical endpoint analyses were therefore interpreted as exploratory and hypothesis-generating. A Firth penalized logistic sensitivity analysis for mortality was also non-significant (direction-aligned OR 1.40, 95% CI 0.29–6.13; *P* = 0.662; Supplementary Table S7D). Subgroup analyses indicated consistent DIDO reductions across key patient categories (Fig. 3C, Supplementary Table S12). Stent implantation and length of hospital stay are reported only as descriptive resource-utilization indicators (Supplementary Fig. S5).

**Table 1:** In-hospital Clinical Outcomes and Segmented Logistic Regression Estimates.

Outcome	Pre-intervention n/N (%)	Post-intervention n/N (%)	Risk difference, % (95% CI)	Unadjusted P	Immediate level OR (95% CI), P	Slope-change OR/month (95% CI), P	Model-based combined OR at 12 months after intervention (95% CI), P	Model-based combined OR at 24 months after intervention (95% CI), P
Composite complications	68/357 (19.05%)	57/428 (13.32%)	−5.73 (−10.92 to −0.54)	0.029	1.756 (0.806–3.826), *P* = 0.157	0.966 (0.926–1.008), *P* = 0.112	1.161 (0.403–3.340), *P* = 0.782	0.767 (0.177–3.323), *P* = 0.723
New-onset heart failure	45/357 (12.61%)	32/428 (7.48%)	−5.13 (−9.38 to −0.88)	0.016	4.643 (1.713–12.586), *P* = 0.003	0.949 (0.897–1.003), *P* = 0.065	2.465 (0.590–10.308), *P* = 0.216	1.309 (0.177–9.684), *P* = 0.792
In-hospital mortality	8/357 (2.24%)	15/428 (3.50%)	1.26 (−1.06 to 3.59)	0.296	0.721 (0.147–3.539), *P* = 0.687	0.899 (0.800–1.010), *P* = 0.074	0.202 (0.015–2.767), *P* = 0.231	0.056 (0.001–2.745), *P* = 0.147

**Note:** Data are presented as n/N (%). Clinical outcomes were assessed during the index hospitalization. Composite complications included heart failure, bleeding, malignant arrhythmia, infection, respiratory failure, mechanical complication, ischemic stroke, renal failure, death, shock, or recurrent myocardial infarction; multiple events in one patient were counted once. Heart failure and death were also analyzed separately as component outcomes. Risk difference was calculated as post-intervention minus pre-intervention. Segmented logistic regression models were specified as logit[P(event)] = β0 + β1 × time + β2 × intervention + β3 × time after intervention. The immediate level OR corresponds to exp.(β2), the slope-change OR/month corresponds to exp.(β3), and the model-based combined ORs at 12 and 24 months after intervention correspond to exp.(β2 + 12β3) and exp.(β2 + 24β3), respectively. These combined ORs are estimates at specified post-intervention calendar times and do not represent patient-level 12- or 24-month follow-up. ORs are reported for event occurrence; therefore, OR > 1 indicates higher odds of the event and OR < 1 indicates lower odds of the event. Robust HC1 standard errors were used for segmented logistic estimates. CI = confidence interval; OR = odds ratio.

**Fig. 3:**
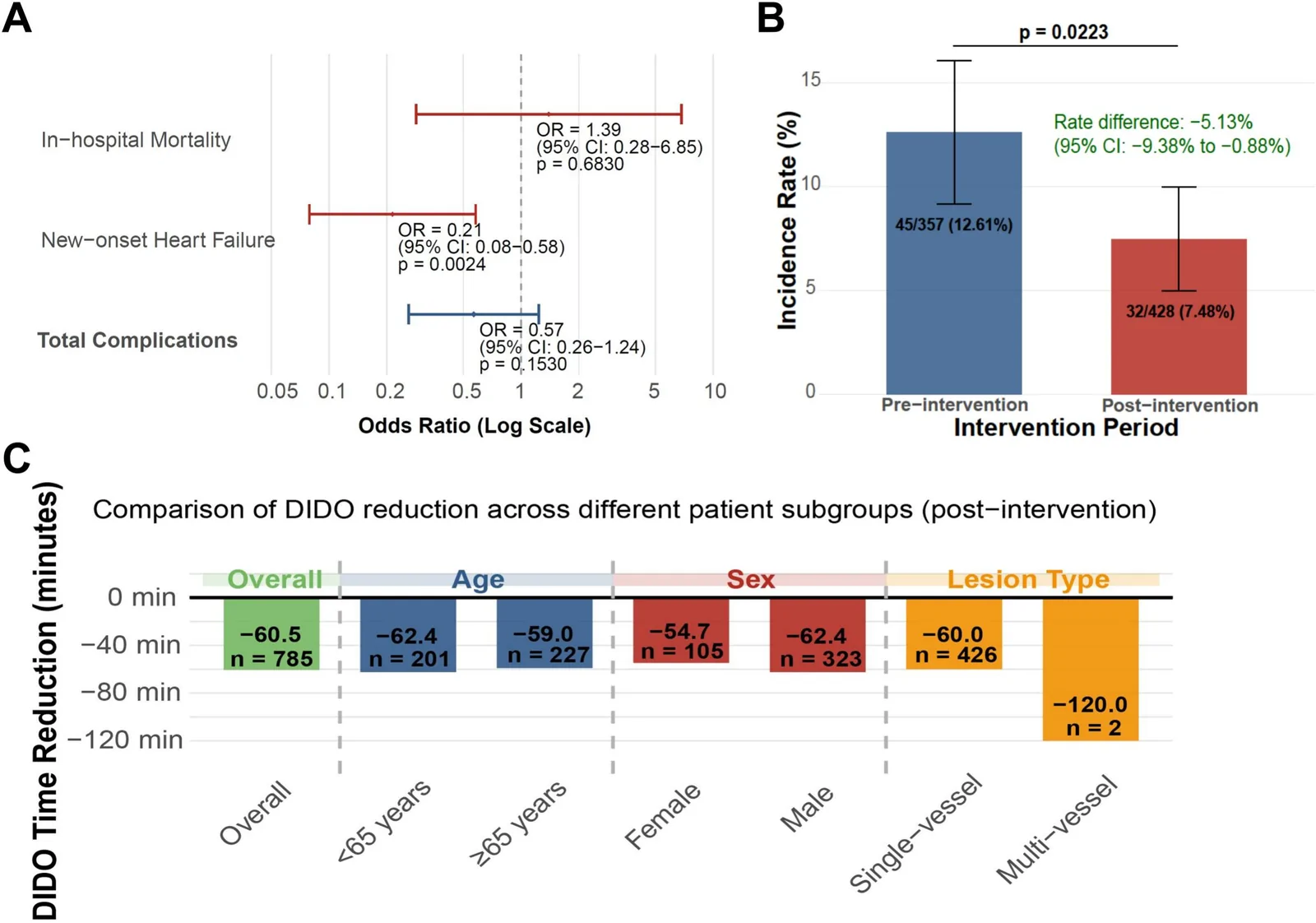
**Clinical outcomes and DIDO subgroup analyses. (A) Forest plot of segmented logistic estimates for in-hospital clinical outcomes. The 12- and 24-month estimates are model-based combined effects at specified calendar times after intervention and do not represent patient follow-up. (B) New-onset in-hospital heart failure before and after intervention. Heart failure is a component of the composite complications endpoint and is also reported separately. (C) Subgroup analysis for DIDO.** CI = confidence interval, DIDO = door-in–door-out, ITS = interrupted time series, OR = odds ratio.

### 3.5 Sensitivity and supplementary analyses

The findings were robust across multiple sensitivity analyses and alternative model specifications. Detailed data cleaning, intervention components, timestamp definitions, ITS coefficients and diagnostics, included-versus-excluded patient comparisons, quarterly clinical outcome trends, and supplementary mechanistic and sensitivity analyses are provided in **Supplementary Tables S1–S12 and Supplementary Figs. S1–S8.**

## 4 Discussion

### 4.1 Summary of findings and system-level impact

In this ITS analysis conducted within a regional hub-and-spoke chest pain center network, implementation of a structured training program in non-PCI-capable units was associated with a significant and sustained reduction in DIDO time. This reduction was accompanied by shorter FMC2W, supporting an association between spoke-side transfer-workflow optimization and shorter FMC2W, a system-level proxy for total ischemic time. Clinical outcome findings were exploratory: crude rates of composite complications and new-onset in-hospital heart failure were lower after the intervention, but full segmented logistic models did not show statistically significant model-based combined effects at 12 or 24 months after the intervention. These estimates refer to modeled calendar time after implementation rather than patient follow-up. The time-efficiency findings align with prior system-level chest pain center studies [14], [15]. The intervention effect remained evident throughout the available post-intervention observation period ending in September 2025, corresponding to approximately 44 months of system-level observation. This persistence applies to the transfer-time outcome and should not be interpreted as long-term patient-level clinical follow-up.

### 4.2 Centralization and operator-to-patient transfer

The present network should be understood as a semi-centralized regional STEMI transfer system rather than a fully centralized triage system. Initial triage, ECG acquisition, STEMI recognition, and transfer initiation occur within each spoke chest pain unit, whereas standardized protocols, training, quality feedback, and early hub notification are coordinated by the PCI-capable hub. The alternative strategy of transferring the interventional operator to the patient was not evaluated in this study. In our setting, spoke units lack catheterization laboratories, PCI equipment, radiation-protection infrastructure, and complete peri-procedural support; therefore, transferring an operator alone would not enable primary PCI at the spoke site and could introduce additional delay. Rapid patient transfer to the PCI-capable hub remains the practical strategy in this regional system, although future research may compare alternative models in settings with different infrastructure.

### 4.3 Mechanistic pathways and workflow stability

Mechanistically, the strong DIDO–FMC2W association suggests that improvements in spoke-side transfer efficiency were accompanied by shorter FMC2W, a system-level proxy for total ischemic time [7], [10]. Associations involving DIDO were stronger than those involving transport duration (D2D) or hub-side in-hospital process times (D2W). Because D2D and hub-side D2W remained comparatively stable and were not direct targets of the intervention, the findings support the interpretation that the observed change was primarily attributable to optimization of spoke-unit workflows rather than to major downstream changes at the PCI-capable hub [7], [10]. The clinical maxim “time is muscle” remains central: the benefit of PCI is tightly coupled to the restoration of coronary flow, yet the logistics of patient delivery often lag behind procedural success [16], [17]. While the intervention achieved a marked and sustained reduction in DIDO, some residual delays likely remain attributable to system-level constraints, such as ambulance availability, cross-facility coordination, and diagnostic turnaround times [18].

Potential alternative explanations should also be considered. To the best of our knowledge, no major contemporaneous change in hub-side catheterization workflow, regional transfer policy, or the institutional PCI pathway occurred at Hanzhong Central Hospital after February 2022. Nevertheless, residual time-varying confounding cannot be fully excluded. Regional EMS availability, post-pandemic healthcare utilization patterns, gradual maturation of the chest pain center network, changes in documentation quality, or other unmeasured quality-improvement activities may have contributed to temporal changes. These factors are partly addressed by the extended ITS design, negative-control analyses, and robustness checks, but they cannot be eliminated in the absence of a parallel control network.

### 4.4 Clinical outcomes and resource utilization

Clinically, crude pre/post comparisons suggested lower rates of composite complications and new-onset in-hospital heart failure after the intervention. However, full segmented logistic models did not provide definitive evidence of sustained clinical-outcome benefit because the model-based combined effects at 12 and 24 months after the intervention were not statistically significant for composite complications, new-onset in-hospital heart failure, or mortality. These values were derived from the fitted segmented models and were not based on longitudinal patient follow-up. Mortality estimates remained imprecise because of low event counts, and the Firth penalized logistic sensitivity analysis also showed no statistically significant mortality change. The clinical endpoint findings should therefore be interpreted as exploratory and hypothesis-generating. Stent implantation and length of hospital stay remained stable and were interpreted only as descriptive resource-utilization indicators; no formal economic evaluation was performed.

### 4.5 Spectral dynamics of transfer efficiency

One novel aspect of this study was the use of spectral analysis to characterize temporal changes in DIDO time dynamics [19]. Prior to the intervention, DIDO times exhibited notable periodic fluctuations likely reflecting systemic factors such as workforce turnover, regional resource allocation, and training cycles. After the intervention, the amplitude of periodic fluctuations decreased and the contribution of dominant periodic components to total variance declined, consistent with an attenuation of cyclical dominance and reduced variability [19]. With irregular fluctuations reduced, an annual component became more clearly discernible, suggesting that residual seasonality accounted for a larger share of the remaining variation [[19], [20]]. These exploratory findings provide complementary support for reduced DIDO variability and suggest that the training-centered workflow optimization may have improved the predictability of the transfer process. Given the limited effective sample size for spectral estimation, these frequency-domain findings should be interpreted as supportive rather than definitive [[19], [20]].

### 4.6 Study limitations

Several limitations of this study must be acknowledged. First, as a single-hub, network-based before–after study without a parallel control group, residual confounding by time-varying or secular changes cannot be entirely excluded [[11], [21]]. Although the extended interrupted time series analysis explicitly modeled underlying temporal trends, it cannot eliminate unmeasured contemporaneous changes [[10], [11], [21]]. Second, registry timestamps may be affected by manual entry error, documentation delay, or imperfect clock synchronization across hospitals; such measurement error could bias interval estimates. Baseline imbalances between study periods may reflect changes in clinical practice and referral patterns, and residual confounding may remain despite time-series modeling and sensitivity analyses. Third, the primary DIDO analysis excluded period-specific extreme values. Comparisons of included and excluded patients and analyses using alternative trimming strategies supported the robustness of the main DIDO result, but selection bias cannot be excluded. Fourth, clinical outcomes were limited to the index hospitalization. The model-based combined effects at 12 and 24 months after the intervention were derived from fitted segmented logistic models and did not represent patient-level follow-up; therefore, the study cannot establish long-term clinical benefit. Mortality and several other clinical endpoints were infrequent, resulting in imprecise estimates and limiting model stability. Fifth, some subgroup analyses had small sample sizes. Sixth, no formal cost-effectiveness analysis was performed because patient-level cost and utility data were unavailable; stent use and length of stay were interpreted only as descriptive resource-utilization indicators. Finally, spectral analyses were complementary and exploratory, and the limited number of monthly observations constrains the precision of frequency-domain inference. Future multicenter controlled studies should incorporate longitudinal patient follow-up, prespecified clinical endpoints, and formal patient-level economic evaluation [[11], [13], [21]].

## 5 Conclusion

In a regional hub-and-spoke chest pain center network, a structured, training-based optimization program at non-PCI chest pain units was associated with a substantial and sustained reduction in DIDO time. This improvement was supported by ITS modeling and autocorrelation-robust sensitivity analyses and was accompanied by shorter FMC2W. Clinical outcome analyses suggested lower crude rates of composite complications and new-onset in-hospital heart failure after the intervention, but full segmented logistic models did not provide definitive evidence of sustained clinical-outcome benefit; mortality estimates remained imprecise because of low event counts. These findings support the feasibility and potential effectiveness of hub-led, training-centered workflow optimization for strengthening regional STEMI transfer systems, while underscoring the need for cautious interpretation of clinical outcomes and validation in multicenter controlled studies.

## CRediT authorship contribution statement

**Xiaolei Ma:** Writing – original draft, Funding acquisition, Formal analysis, Data curation, Conceptualization. **Xianjun Wu:** Writing – review & editing, Supervision. **Liang Wen:** Writing – review & editing, Validation. **Ruifeng Wang:** Validation, Formal analysis. **Jian Cao:** Validation, Formal analysis. **Tong Zhang:** Investigation, Data curation. **Kun Bai:** Investigation, Data curation. **Zhongshang Guo:** Visualization, Supervision, Methodology, Conceptualization. **Yi Xie:** Supervision, Resources, Project administration.

## Institutional review board statement

This study was approved by the Ethics Committee of Hanzhong Central Hospital (Approval No.: 2025-Lun-Wei-Shen-128). This is a retrospective study utilizing archival clinical data. All patient data were fully de-identified and anonymized prior to analysis to protect privacy; no personally identifiable information was accessed or used. Given that this research involves only the analysis of existing, anonymized data without any direct intervention or contact with participants, it poses no risk to patient privacy or safety. Therefore, in accordance with relevant ethical guidelines and regulations, the requirement for informed consent was waived by the Ethics Committee.

## Funding

This study was funded by the Internal Research Grant from Hanzhong Central Hospital (No. YK1919).

## Declaration of competing interest

The authors declare that they have no known competing financial interests or personal relationships that could have appeared to influence the work reported in this paper.

## Data Availability

The datasets used and/or analyzed during the current study are derived from the Chest Pain Center Data Reporting Platform, a national registry managed by relevant health authorities. Due to strict data privacy regulations and the proprietary nature of this national database, the raw data are not publicly available. Access to the data is restricted and requires formal approval from the platform's governing body. Researchers wishing to access similar data for verification or further research must submit a formal application directly to the Chest Pain Center Data Reporting Platform administration in accordance with their data sharing policies.
