# Supplementary material for: Transfer-workflow optimization in a regional chest pain center network reduces door-in–door-out time in ST-segment elevation myocardial infarction: An interrupted time-series study with spectral validation
Source: Int J Cardiol Heart Vasc. 2026 Sep 25;67:102020. doi: 10.1016/j.ijcha.2026.102020 (PMC13635709; doi:10.1016/j.ijcha.2026.102020)
Supplement: Supplementary file 1 [file mmc1.docx]

**Supplementary Material**

**Transfer-Workflow Optimization in a Regional Chest Pain Center Network Reduces Door-In–Door-Out Time in ST-Segment Elevation Myocardial Infarction: An Interrupted Time-Series Study with Spectral Validation**

Xiaolei Ma ^a^, Xianjun Wu ^a^, Liang Wen ^a^, Ruifeng Wang ^a^, Jian Cao ^a^, Tong Zhang ^a^, Kun Bai ^a^, Zhongshang Guo ^b, *^, Yi Xie ^a, *^

^a^ *Coronary Care Unit (CCU), Hanzhong Central Hospital, Hanzhong, 723000, China*

*^b^ Department of Osteoarticular Surgery, Hanzhong Central Hospital, Hanzhong, 723000, China*

**Corresponding authors**

Zhongshang Guo, Department of Osteoarticular Surgery, Hanzhong Central Hospital, No. 557 Middle Laodong West Road, Hantai District, Hanzhong, Shaanxi 723000, China

Email: guozhongshang001@163.com

Yi Xie, Coronary Care Unit (CCU), Hanzhong Central Hospital, No. 557 Middle Laodong West Road, Hantai District, Hanzhong, Shaanxi 723000, China

Email: 19392071@qq.com

**Supplementary Methods**

Study setting and network organization. Hanzhong Central Hospital is the PCI-capable regional hub and tertiary referral center for the local chest pain center network in Hanzhong, Shaanxi Province, China. The affiliated spoke sites are chest pain units without on-site catheterization laboratory or primary PCI capability. Therefore, STEMI patients initially presenting to these non-PCI-capable units require inter-hospital transfer to the hub for coronary angiography and PCI. The network operates as a semi-centralized system: standardized protocols, training, quality feedback, and hub-notification pathways are coordinated by the hub, whereas initial triage, STEMI recognition, first ECG/laboratory testing, and transfer initiation are performed locally within each spoke unit.

 Data extraction and quality control. De-identified structured registry records were obtained from the National Chest Pain Center Reporting Platform for all eligible transferred STEMI cases during the study period. Data extraction was based on predefined platform fields and automated structured-data processing. All cases within the predefined time window and regional network were included without subjective case selection. Field mapping, derived time intervals, and logical consistency were checked before analysis. Formal blinding to the study hypothesis was not feasible because of the retrospective registry-based design; however, the use of predefined standardized platform fields minimized subjective outcome ascertainment.

 Timestamp definitions and interval derivation. All time intervals were calculated directly from two corresponding standardized platform timestamp fields. DIDO was calculated as departure time from the spoke hospital minus arrival time at the spoke hospital. D2D was calculated as arrival time at the hub minus departure time from the spoke hospital. D2W was calculated as wire-crossing time minus arrival time at the hub. FMC2W was calculated as wire-crossing time minus first medical contact time. No subjective selection among competing timestamp candidates was required.

 Outlier management. To minimize the influence of extreme transfer delays attributable to atypical clinical pathways, including patients transferred after prolonged stays in a chest pain unit, individual observations with DIDO values above the period-specific 95th percentile were excluded before calculation of monthly means. This trimming strategy excluded 42 of 827 records and yielded 785 patients for the primary analysis. Included and excluded patients were compared using descriptive statistics and standardized mean differences.

 Interrupted time-series diagnostics and robust inference. The primary segmented regression model used monthly mean DIDO as the dependent variable and included terms for the baseline time trend, the immediate post-intervention level change, and the post-intervention slope change. Residual autocorrelation was evaluated using Durbin-Watson and Ljung-Box tests, with residual ACF/PACF inspection. Because diagnostic tests suggested residual serial correlation, Newey-West robust standard errors and a generalized least-squares model with AR(1) correlation structure were used as sensitivity analyses. The immediate DIDO reduction remained directionally consistent across OLS-HC1, Newey-West, and GLS-AR1 specifications.

 Clinical outcome definitions. Clinical outcomes were assessed during the index hospitalization, from admission until discharge or in-hospital death. The registry-defined composite complications endpoint was present if at least one of the following occurred: heart failure, bleeding, malignant arrhythmia, infection, respiratory failure, mechanical complication, ischemic stroke, renal failure, death, shock, or recurrent myocardial infarction. Multiple complications in one patient were counted once. Heart failure and death were also analyzed separately as clinically relevant component outcomes.

 Clinical outcome trend and low-frequency endpoint sensitivity. Quarterly event counts and rates were summarized for composite complications, new-onset in-hospital heart failure, and in-hospital mortality. Because mortality was infrequent, a Firth penalized logistic sensitivity model was additionally used to evaluate the robustness of mortality inference. The Firth sensitivity analysis did not show a statistically significant immediate post-intervention level change in mortality.

 Mechanistic and specificity analyses. The association between monthly mean DIDO and FMC2W was evaluated using Pearson correlation with 95% confidence intervals and Spearman rank correlation as a sensitivity measure. To assess whether this association differed between periods, a DIDO-by-period interaction term was fitted. DIDO versus D2D was evaluated as a negative-control association to support specificity of the transfer-workflow effect.

 Software. Analyses were performed using R version 4.3.3. Time-series models, robust standard errors, generalized least-squares models, Firth logistic regression, and graphics were implemented using base R and relevant packages including lmtest, sandwich, nlme, logistf, dplyr, ggplot2, and related tidyverse utilities.

**Supplementary Table S1. Components of the transfer-workflow optimization intervention**

| Domain | Pre-intervention problem | Optimization measure | Responsible site | Expected effect on DIDO |
| --- | --- | --- | --- | --- |
| Early STEMI recognition | Delayed ECG interpretation or delayed recognition of STEMI at spoke units | Hands-on training in STEMI recognition, standardized ECG interpretation, and early activation criteria | Spoke units | Shorten time from arrival to diagnosis and transfer decision |
| Dedicated triage and reception | Unclear responsibility for chest pain reception and triage | Dedicated reception/triage roles for chest pain patients and enhanced in-hospital signage | Spoke units | Shorten time from door-in to first assessment |
| Internal spoke-unit pathway | Waiting for ECG, laboratory testing, consultation, or administrative procedures | Green-channel workflow, rapid ECG/laboratory testing, simplified internal routing, and predefined transfer checklist | Spoke units | Reduce avoidable in-hospital holding time before transfer |
| Referral activation and hub notification | Delayed hub notification, non-standardized transfer communication, or patient self-transfer | Earlier hub notification, standardized referral contact pathway, and transfer-bypass process | Spoke units + Hub | Shorten pre-departure waiting and improve coordination |
| Hub preparation and downstream care | Hub PCI workflow was not the target of the intervention | Existing hub catheterization-laboratory pathway was maintained without structural change during the study period | Hub | Provide specificity context; downstream hub processes served as non-targeted comparators |

**Note**: The intervention was designed to optimize spoke-side processes that determine DIDO time. The hub-side PCI workflow was not the intervention target.

**Supplementary Table S2. Operational definitions of registry timestamps and derived intervals**

| **Timestamp or interval** | **Operational definition** | **Use in analysis** |
| --- | --- | --- |
| Symptom onset time | Patient-reported time of onset of ischemic symptoms recorded in the registry | Descriptive baseline timing variable |
| First medical contact (FMC) | Time at which the patient first received medical evaluation within the care pathway | Starting timestamp for FMC2W |
| Arrival at spoke hospital | Door-in time at the non-PCI-capable chest pain unit / referring hospital | Starting timestamp for DIDO |
| Departure from spoke hospital | Door-out time from the non-PCI-capable chest pain unit / referring hospital | Ending timestamp for DIDO and starting timestamp for D2D |
| Arrival at hub hospital | Door-in time at Hanzhong Central Hospital, the PCI-capable regional hub | Ending timestamp for D2D and starting timestamp for D2W |
| Wire-crossing time | Time at which the guidewire crossed the culprit coronary lesion during PCI | Ending timestamp for D2W and FMC2W |
| DIDO | Departure from spoke hospital minus arrival at spoke hospital | Primary transfer-efficiency outcome |
| D2D | Arrival at hub hospital minus departure from spoke hospital | Transport interval and negative-control downstream interval |
| D2W | Wire-crossing time minus arrival at hub hospital | Hub-side in-hospital PCI process interval |
| FMC2W | Wire-crossing time minus first medical contact time | Total ischemic-time proxy and mechanistic downstream outcome |

**Note:** Intervals were derived directly from standardized timestamp fields. No multiple competing timestamp fields required subjective selection.

**Supplementary Table S3. Detailed Data Cleaning and Outlier Management Report**

| **Study Phase** | **Initial Enrolled Population (**$n$**)** | **Excluded Outliers (**$n$**)** | **Final Analytical Population (**$n$**)** |
| --- | --- | --- | --- |
| **Pre-intervention** | 376 | 19 | 357 |
| **Post-intervention** | 451 | 23 | 428 |
| **Total** | **827** | **42** | **785** |

**Note**: To ensure the robustness of the time-series analysis and minimize the influence of extreme transfer delays, individual observations with DIDO values exceeding the 95th percentile within each period (pre- and post-intervention) were excluded prior to calculating monthly means. This a priori trimming resulted in the exclusion of 19 pre-intervention and 23 post-intervention observations, yielding a final analytical cohort of 785 patients. To validate this approach, prespecified sensitivity analyses were performed using alternative thresholds (e.g., 90th and 97th percentiles) and an untrimmed dataset. Results demonstrated that the estimated intervention effects on DIDO time remained materially unchanged and directionally consistent across all specifications, supporting the stability of the primary analytical findings. DIDO = door-in–door-out.

**Supplementary Table S4. ITS diagnostics and autocorrelation-robust sensitivity analyses for monthly mean DIDO**

**A. Autocorrelation diagnostics and model fit**

| Metric | Value |
| --- | --- |
| Durbin-Watson statistic | 1.685 |
| Durbin-Watson P | 0.043 |
| Ljung-Box statistic | 26.454 |
| Ljung-Box P | 0.009 |
| AIC (OLS) | 679.95 |
| BIC (OLS) | 691.26 |
| AIC (GLS AR1) | 680.39 |
| BIC (GLS AR1) | 693.97 |

**B. Immediate post-intervention level-change estimates across ITS specifications**

| Model | Estimate, min | SE | 95% CI | P value |
| --- | --- | --- | --- | --- |
| OLS_HC1 | -49.57 | 18.45 | -85.72 to -13.41 | 0.009 |
| OLS_NeweyWest | -49.57 | 14.83 | -78.63 to -20.51 | 0.001 |
| GLS_AR1 | -49.33 | 15.59 | -79.90 to -18.77 | 0.002 |

**Note**: OLS-HC1 used heteroskedasticity-consistent standard errors; Newey-West used autocorrelation-robust standard errors; GLS-AR1 used a generalized least-squares model with AR(1) residual correlation. Negative estimates indicate shorter DIDO time after the intervention.

**Supplementary Table S5. Full segmented logistic regression estimates for binary in-hospital clinical outcomes**

| Outcome | Model quantity | log(OR) | Robust SE | OR (95% CI) | P value |
| --- | --- | --- | --- | --- | --- |
| Composite complications | Immediate level effect | 0.563 | 0.397 | 1.756 (0.806–3.826) | 0.157 |
|  | Post-intervention slope change, per month | −0.035 | 0.022 | 0.966 (0.926–1.008) | 0.112 |
|  | Model-based combined effect at 12 months after intervention | 0.149 | 0.539 | 1.161 (0.403–3.340) | 0.782 |
|  | Model-based combined effect at 24 months after intervention | −0.265 | 0.748 | 0.767 (0.177–3.323) | 0.723 |
| New-onset in-hospital heart failure | Immediate level effect | 1.535 | 0.509 | 4.643 (1.713–12.586) | 0.003 |
|  | Post-intervention slope change, per month | −0.052 | 0.028 | 0.949 (0.897–1.003) | 0.065 |
|  | Model-based combined effect at 12 months after intervention | 0.902 | 0.730 | 2.465 (0.590–10.308) | 0.216 |
|  | Model-based combined effect at 24 months after intervention | 0.269 | 1.021 | 1.309 (0.177–9.684) | 0.792 |
| In-hospital mortality | Immediate level effect | −0.327 | 0.812 | 0.721 (0.147–3.539) | 0.687 |
|  | Post-intervention slope change, per month | −0.106 | 0.059 | 0.899 (0.800–1.010) | 0.074 |
|  | Model-based combined effect at 12 months after intervention | −1.599 | 1.331 | 0.202 (0.015–2.767) | 0.231 |
|  | Model-based combined effect at 24 months after intervention | −2.882 | 2.020 | 0.056 (0.001–2.745) | 0.147 |

**Note:** Segmented logistic regression models were specified as:

logit[P(event)] = β0 + β1 × time + β2 × intervention + β3 × time after intervention.

Clinical outcomes were assessed during the index hospitalization. Composite complications included heart failure, bleeding, malignant arrhythmia, infection, respiratory failure, mechanical complication, ischemic stroke, renal failure, death, shock, or recurrent myocardial infarction; multiple events in one patient were counted once. Heart failure and death were also analyzed separately as component outcomes. The immediate level effect corresponds to exp(β2), and the post-intervention slope-change effect corresponds to exp(β3). The model-based combined effects at 12 and 24 months after intervention were calculated as exp(β2 + 12β3) and exp(β2 + 24β3), respectively. These estimates refer to the modeled intervention effect at specified points in calendar time after implementation and do not represent outcomes after 12 or 24 months of patient follow-up. ORs are reported for event occurrence; therefore, OR >1 indicates higher odds of the event and OR <1 indicates lower odds of the event. Robust HC1 standard errors were used. Although crude pre/post comparisons showed lower overall rates of composite complications and new-onset in-hospital heart failure after the intervention, the model-based combined effects were not statistically significant. These clinical endpoint analyses should therefore be interpreted as exploratory and hypothesis-generating rather than definitive evidence of clinical benefit. CI = confidence interval; OR = odds ratio; SE = standard error.

**Supplementary Table S6A. Comparison of Dominant Periodic Components Before and After Intervention**

*(This table details the changes in the spectral characteristics of the time series, highlighting the shift in dominant frequencies and reduction in amplitude.)*

| **Rank** | **Period (months)** | **Amplitude (min)** | **Variance Explained (%)** |
| --- | --- | --- | --- |
| **Pre-intervention** |  |  |  |
| 1 | 64.0 | 3044.8 | 69.8 |
| 2 | 21.3 | 1038.3 | 8.1 |
| 3 | 16.0 | 899.5 | 6.1 |
| **Post-intervention** |  |  |  |
| 1 | 64.0 | 1570.9 | 55.0 |
| 2 | 32.0 | 933.6 | 19.4 |
| 3 | 12.8 | 521.5 | 6.1 |

**Note:** “Rank” denotes the ordering of spectral components by their contribution to total variance. “Variance explained” represents the proportion of the signal energy attributable to a given periodicity. A reduction in the amplitude of the dominant component (Rank 1) indicates attenuation of cyclical dominance and reduced overall variability in DIDO time after the intervention. DIDO = door-in–door-out.

**Supplementary Table S6B. Sensitivity Analyses and Robustness Assessment of Spectral Estimation**

*(This table summarizes the stability of the spectral analysis results under various methodological perturbations.)*

| **Analysis Method** | **Metric** | **Pre-intervention Value** | **Post-intervention Value** | **Stability Assessment** |
| --- | --- | --- | --- | --- |
| **Zero-padding** | Cycle CV (%) | 108.8 | 105.9 | Unstable |
| **Bootstrap** | 95% CI Mean Width | 262.02 | 173.37 | Unstable |
| **Jackknife** | Mean CV (%) | 0.5 | 0.0 | **Stable** |
| **Temporal Offset** | Period Difference (months) | 0.0–0.0 | 0.0–0.0 | **Stable** |
| **Overall Score** | **Total Robustness (0–4)** |  |  | **2 (Moderate)** |

**Note:** Robustness was assessed across four complementary dimensions. One point was assigned for each method classified as “Stable.” An overall score of 2/4 indicates moderate robustness, supported by high stability in the Jackknife and temporal offset analyses, whereas greater variability was observed in the bootstrap and zero-padding approaches, likely reflecting limitations in effective sample size. CV = coefficient of variation, CI = confidence interval.

**Supplementary Table S7. DIDO-FMC2W association, negative-control analysis, and small-sample mortality sensitivity**

**A. Correlation between monthly mean DIDO and FMC2W**

| Group | Method | N | Correlation coefficient | 95% CI | P value |
| --- | --- | --- | --- | --- | --- |
| Overall | Pearson | 71 | 0.921 | 0.876 to 0.950 | <0.001 |
| Pre-intervention | Pearson | 27 | 0.957 | 0.907 to 0.980 | <0.001 |
| Post-intervention | Pearson | 44 | 0.755 | 0.591 to 0.859 | <0.001 |
| Overall | Spearman | 71 | 0.899 | - | <0.001 |
| Pre-intervention | Spearman | 27 | 0.922 | - | <0.001 |
| Post-intervention | Spearman | 44 | 0.798 | - | <0.001 |

**B. DIDO-by-period interaction model for FMC2W**

| Term | Estimate | SE | 95% CI | P value |
| --- | --- | --- | --- | --- |
| (Intercept) | 72.777 | 8.653 | 55.816 to 89.737 | <0.001 |
| mean_DIDO | 1.065 | 0.049 | 0.969 to 1.162 | <0.001 |
| post | 23.148 | 15.420 | -7.074 to 53.371 | 0.138 |
| mean_DIDO:post | -0.096 | 0.142 | -0.374 to 0.182 | 0.502 |

**C. Negative-control association between monthly mean DIDO and D2D**

| Pair | N | Pearson r | 95% CI | P value |
| --- | --- | --- | --- | --- |
| DIDO vs D2D | 71 | -0.061 | -0.290 to 0.175 | 0.614 |

**D. Firth penalized logistic sensitivity analysis for mortality**

| Parameter | Direction-aligned OR | 95% CI | P value |
| --- | --- | --- | --- |
| Immediate level change for mortality (direction-aligned with main Table 1) | 1.40 | 0.29 to 6.13 | 0.662 |

**Note**: ORs in panel D were direction-aligned to match the event-occurrence interpretation used in the main segmented logistic regression table. The original Firth model output for the reciprocal contrast was inverted for consistency. DIDO = door-in-door-out; D2D = door-to-door; FMC2W = first medical contact-to-wire crossing.

**Supplementary Table S8. Baseline Characteristics and Clinical Data**

| **Variable** | **Overall (n=785)** | **Pre-intervention (n=357)** | **Post-intervention (n=428)** | **P value** |
| --- | --- | --- | --- | --- |
| **Demographics** |  |  |  |  |
| Age (years), mean ± SD | 63.7 ± 12.2 | 62.8 ± 12.0 | 64.5 ± 12.3 | **0.046** |
| Male, n (%) | 598 (76.2%) | 275 (77.0%) | 323 (75.5%) | 0.669 |
| Occupation (Farmer), n (%) | 387 (49.3%) | 230 (64.4%) | 157 (36.7%) | **<0.001** |
| **Clinical Characteristics** |  |  |  |  |
| Symptom to FMC (min), M [IQR] | 206 [95-720] | 202 [100-740] | 209 [90-698] | 0.991 |
| Persistent Chest Pain, n (%) | 620 (79.0%) | 284 (79.6%) | 336 (78.5%) | 0.787 |
| Killip Classification, n (%) |  |  |  |  |
| Class I (no CHF) | 672 (86.4%) | 301 (85.5%) | 371 (87.1%) | 0.594 |
| Class II (rales/JVD) | 69 (8.9%) | 32 (9.1%) | 37 (8.7%) | 0.943 |
| Class III (pulmonary edema) | 11 (1.4%) | 10 (2.8%) | 1 (0.2%) | **0.003** |
| Class IV (cardiogenic shock) | 26 (3.3%) | 9 (2.6%) | 17 (4.0%) | 0.364 |
| LVEF (%), mean ± SD | 54.3 ± 9.5 | 56.3 ± 9.8 | 52.6 ± 8.9 | **<0.001** |
| **Laboratory Tests** |  |  |  |  |
| CK-MB ≥5.0 ng/ml, n (%) | 707 (92.3%) | 347 (97.5%) | 360 (87.8%) | **<0.001** |
| D-dimer (ng/mL), M [IQR] | 0.4 [0.2-0.7] | 0.4 [0.2-0.8] | 0.3 [0.2-0.7] | 0.062 |
| NT-proBNP (pg/mL), M [IQR] | 1065.1 [379-3093] | 714.3 [318-2388] | 1413.6 [465-3855] | **<0.001** |
| Positive Troponin, n (%) | 612 (79.7%) | 316 (89.0%) | 296 (71.7%) | **<0.001** |
| Creatinine (ng/mL), M [IQR] | 65.8 [54.0-82.0] | 62.8 [52.2-77.5] | 69.0 [56.0-86.0] | **<0.001** |
| Total Cholesterol (mmol/L), M [IQR] | 4.1 [3.5-4.8] | 4.1 [3.4-4.8] | 4.2 [3.6-4.7] | 0.547 |
| Triglycerides (mmol/L), M [IQR] | 1.3 [0.9-2.0] | 1.3 [0.9-1.9] | 1.4 [0.9-2.0] | 0.051 |
| HDL-C (mmol/L), M [IQR] | 1.1 [0.9-1.3] | 1.1 [0.9-1.3] | 1.1 [0.9-1.3] | 0.122 |
| LDL-C (mmol/L), M [IQR] | 2.5 [2.0-3.0] | 2.4 [1.9-3.0] | 2.5 [2.0-3.0] | 0.408 |
| **Risk Factors, n (%)** |  |  |  |  |
| Smoking | 466 (59.4%) | 228 (63.9%) | 238 (55.6%) | **0.023** |
| Family History of Premature CVD | 8 (1.0%) | 5 (1.4%) | 3 (0.7%) | 0.479 |
| Diabetes | 194 (24.7%) | 80 (22.4%) | 114 (26.6%) | 0.199 |
| Overweight/Obesity | 51 (6.5%) | 21 (5.9%) | 30 (7.0%) | 0.622 |
| Dyslipidemia | 181 (23.1%) | 75 (21.0%) | 106 (24.8%) | 0.246 |
| Hypertension | 412 (52.5%) | 199 (55.7%) | 213 (49.8%) | 0.110 |
| **Comorbidities, n (%)** |  |  |  |  |
| COPD | 13 (1.7%) | 5 (1.4%) | 8 (1.9%) | 0.817 |
| Aortic Aneurysm | 1 (0.1%) | 1 (0.3%) | 0 (0.0%) | 0.455 |
| Coronary Artery Disease | 114 (14.5%) | 65 (18.2%) | 49 (11.4%) | **0.010** |
| Peripheral Arterial Disease | 4 (0.5%) | 2 (0.6%) | 2 (0.5%) | 1.000 |
| Atrial Fibrillation | 50 (6.4%) | 21 (5.9%) | 29 (6.8%) | 0.716 |
| Valvular Heart Disease | 51 (6.5%) | 14 (3.9%) | 37 (8.6%) | **0.011** |
| Malignancy | 7 (0.9%) | 2 (0.6%) | 5 (1.2%) | 0.464 |
| Chronic Heart Failure | 6 (0.8%) | 3 (0.8%) | 3 (0.7%) | 1.000 |
| Chronic Kidney Disease | 20 (2.5%) | 6 (1.7%) | 14 (3.3%) | 0.238 |
| Peptic Ulcer | 6 (0.8%) | 3 (0.8%) | 3 (0.7%) | 1.000 |
| Thyroid Dysfunction | 63 (8.0%) | 20 (5.6%) | 43 (10.0%) | **0.032** |
| Cerebrovascular Disease | 51 (6.5%) | 17 (4.8%) | 34 (7.9%) | 0.098 |
| Anemia | 21 (2.7%) | 11 (3.1%) | 10 (2.3%) | 0.673 |
| **Treatment** |  |  |  |  |
| Door-to-Wire Time (min), M [IQR] | 34 [27-52] | 31 [26-47] | 37 [29-55] | **<0.001** |
| Single Vessel Disease, n (%) | 779 (99.2%) | 353 (98.9%) | 426 (99.5%) | 0.419 |
| Multi-vessel Disease, n (%) | 6 (0.8%) | 4 (1.1%) | 2 (0.5%) | 0.419 |
| Total Stents Implanted, mean ± SD | 1.1 ± 0.6 | 1.1 ± 0.6 | 1.1 ± 0.6 | 0.999 |
| **Discharge Information** |  |  |  |  |
| Length of Stay (days), M [IQR] | 4 [3-4] | 4 [4-4] | 4 [3-4] | **0.012** |
| Improvement, n (%) | 723 (92.1%) | 334 (93.6%) | 389 (90.9%) | 0.212 |

**Note:** Data are presented as mean ± standard deviation (SD) for normally distributed continuous variables, median [interquartile range (IQR)] for non-normally distributed continuous variables, and n (%) for categorical variables. P values compare pre-intervention versus post-intervention groups and were calculated using the Mann–Whitney U test for continuous variables and the χ² test or Fisher’s exact test for categorical variables, as appropriate. Percentages may not sum to 100% because of rounding. Descriptive comparisons in this table were performed at the individual-patient level using conventional cross-sectional tests to summarize pre- and post-intervention distributions. In contrast, the primary analysis utilized ITS segmented regression of monthly aggregated metrics to estimate post-intervention changes while accounting for underlying temporal trends. Therefore, statistical significance in this table does not necessarily imply a structural intervention effect; apparent differences may reflect case-mix shifts or temporal variation rather than changes attributable to the intervention. Specifically, while D2W time showed a nominal difference in individual-level comparisons, the ITS analysis found no evidence of a sustained post-intervention level or slope change. This discrepancy suggests that the cross-sectional difference likely reflects short-term fluctuations or case-mix shifts rather than a durable change in downstream hub workflows. Accordingly, we interpret these results as descriptive and base all conclusions regarding intervention impact and specificity on the time-series estimates rather than these cross-sectional P values. CHF = congestive heart failure, COPD = chronic obstructive pulmonary disease, CK-MB = creatine kinase–MB, CVD = cardiovascular disease, HDL-C = high-density lipoprotein cholesterol, LDL-C = low-density lipoprotein cholesterol, LVEF = left ventricular ejection fraction, JVD = jugular venous distension, NT-proBNP = N-terminal pro–B-type natriuretic peptide, D2W = door-to-wire crossing time, DIDO = door-in–door-out, ITS = interrupted time series.

**Supplementary Table S9. Baseline characteristics of included versus excluded patients after DIDO trimming**

| Variable | Included (n=785) | Excluded (n=42) | SMD | P value |
| --- | --- | --- | --- | --- |
| age | 63.70 ± 12.22 | 64.31 ± 11.48 | -0.052 | 0.739 |
| male = Yes | 598 (76.2%) | 33 (78.6%) | -0.057 | 0.866 |
| post_period = Pre | 357 (45.5%) | 19 (45.2%) | -0.005 | 1.000 |
| D2D | 47.57 ± 30.11 | 53.31 ± 29.49 | -0.193 | 0.226 |
| D2W | 43.44 ± 24.70 | 45.71 ± 29.69 | -0.083 | 0.628 |
| FMC2W | 209.86 ± 117.24 | 1683.00 ± 2193.79 | -0.948 | <0.001 |
| heart_failure = Yes | 77 (9.8%) | 3 (7.1%) | -0.096 | 0.789 |
| death = Yes | 23 (2.9%) | 0 (0.0%) | 0.244 | 0.625 |
| complication = Yes | 125 (15.9%) | 4 (9.5%) | -0.192 | 0.371 |
| vessel_n | 1.01 ± 0.09 | 1.00 ± 0.00 | 0.124 | 0.014 |
| stent_n | 1.13 ± 0.57 | 1.14 ± 0.52 | -0.031 | 0.841 |
| los | 4.23 ± 3.19 | 5.57 ± 7.68 | -0.228 | 0.267 |

**Note**: Excluded observations were defined by period-specific extreme DIDO values. Longer FMC2W among excluded patients was expected by design because DIDO is a component of FMC2W. SMD = standardized mean difference.

**Supplementary Table S10. Detailed Parameter Estimates of the ITS Model**

| **Model Parameters** | **Coefficient (**$\beta$**)** | **Standard Error (SE)** | $t$ **statistic** | **P value** |
| --- | --- | --- | --- | --- |
| **Baseline Intercept (**$\beta_{0}$**)** | 163.38 | 10.36 | 15.78 | **<0.001** |
| **Pre-intervention Trend (**$\beta_{1}$**)** | -0.53 | 0.92 | -0.58 | 0.564 |
| **Immediate Level Change (**$\beta_{2}$**)** | -49.66 | 18.47 | -2.69 | **0.009** |
| **Post-intervention Slope Change (**$\beta_{3}$**)** | 0.27 | 0.95 | 0.28 | 0.778 |

**Note:** This table provides a summary of the regression model parameters, with further details on the model specification provided in the Methods section.

**Supplementary Table S11. Quarterly clinical outcomes**

| Quarter | N | Composite complications | New-onset heart failure | In-hospital mortality | Phase |
| --- | --- | --- | --- | --- | --- |
| 2019-Q4 | 40 | 11 (27.5%) | 11 (27.5%) | 1 (2.5%) | Pre-intervention |
| 2020-Q1 | 38 | 6 (15.8%) | 6 (15.8%) | 0 (0.0%) | Pre-intervention |
| 2020-Q2 | 36 | 6 (16.7%) | 3 (8.3%) | 0 (0.0%) | Pre-intervention |
| 2020-Q3 | 41 | 8 (19.5%) | 4 (9.8%) | 0 (0.0%) | Pre-intervention |
| 2020-Q4 | 62 | 10 (16.1%) | 4 (6.5%) | 1 (1.6%) | Pre-intervention |
| 2021-Q1 | 42 | 10 (23.8%) | 6 (14.3%) | 2 (4.8%) | Pre-intervention |
| 2021-Q2 | 28 | 5 (17.9%) | 5 (17.9%) | 0 (0.0%) | Pre-intervention |
| 2021-Q3 | 21 | 1 (4.8%) | 1 (4.8%) | 0 (0.0%) | Pre-intervention |
| 2021-Q4 | 37 | 10 (27.0%) | 5 (13.5%) | 3 (8.1%) | Pre-intervention |
| 2022-Q1 | 34 | 6 (17.6%) | 4 (11.8%) | 1 (2.9%) | Intervention quarter |
| 2022-Q2 | 35 | 8 (22.9%) | 7 (20.0%) | 1 (2.9%) | Post-intervention |
| 2022-Q3 | 26 | 7 (26.9%) | 5 (19.2%) | 2 (7.7%) | Post-intervention |
| 2022-Q4 | 22 | 3 (13.6%) | 2 (9.1%) | 0 (0.0%) | Post-intervention |
| 2023-Q1 | 28 | 6 (21.4%) | 5 (17.9%) | 3 (10.7%) | Post-intervention |
| 2023-Q2 | 26 | 6 (23.1%) | 3 (11.5%) | 2 (7.7%) | Post-intervention |
| 2023-Q3 | 25 | 0 (0.0%) | 0 (0.0%) | 0 (0.0%) | Post-intervention |
| 2023-Q4 | 15 | 2 (13.3%) | 1 (6.7%) | 1 (6.7%) | Post-intervention |
| 2024-Q1 | 47 | 7 (14.9%) | 4 (8.5%) | 2 (4.3%) | Post-intervention |
| 2024-Q2 | 44 | 4 (9.1%) | 1 (2.3%) | 1 (2.3%) | Post-intervention |
| 2024-Q3 | 25 | 2 (8.0%) | 0 (0.0%) | 0 (0.0%) | Post-intervention |
| 2024-Q4 | 35 | 3 (8.6%) | 0 (0.0%) | 1 (2.9%) | Post-intervention |
| 2025-Q1 | 39 | 2 (5.1%) | 0 (0.0%) | 2 (5.1%) | Post-intervention |
| 2025-Q2 | 22 | 1 (4.5%) | 0 (0.0%) | 0 (0.0%) | Post-intervention |
| 2025-Q3 | 17 | 1 (5.9%) | 0 (0.0%) | 0 (0.0%) | Post-intervention |

**Note:** 2022-Q1 was labelled as the intervention quarter because the intervention was implemented in February 2022 and the quarter includes both pre- and post-intervention months.

**Supplementary Table S12. Subgroup Analyses of the Intervention Effect on DIDO Time**

| **Subgroup Category** | **Pre-intervention (**$n$**)** | **Post-intervention (**$n$**)** | **Total (**$N$**)** | **Estimated DIDO Change (min)** |
| --- | --- | --- | --- | --- |
| **Age** |  |  |  |  |
| < 65 years | 185 | 201 | 386 | -62.4 |
| ≥ 65 years | 172 | 227 | 399 | -59.0 |
| **Sex** |  |  |  |  |
| Female | 82 | 105 | 187 | -54.7 |
| Male | 275 | 323 | 598 | -62.4 |
| **Coronary Artery Disease** |  |  |  |  |
| Single-vessel Disease | 353 | 426 | 779 | -60.0 |
| Multi-vessel Disease${}^{\dagger}$ | 4 | 2 | 6 | -120.0 |

**Note:** “Estimated DIDO change” denotes the subgroup-specific reduction in DIDO time associated with the intervention, expressed as an adjusted mean difference (minutes).

† Caution: The analysis for the multi-vessel disease subgroup is based on a very small sample (n = 6 total). The large estimated reduction (−120.0 min) should be interpreted with extreme caution, as it is likely unstable and may not be statistically reliable compared with estimates in other subgroups. DIDO = door-in–door-out time.

**Supplementary Figure S1. Distribution of monthly case volumes.** The bar chart displays the number of STEMI patients included in the analysis for each of the 71 months. Monthly case volume ranged from a minimum of 3 to a maximum of 27 patients. Due to the presence of months with low case volumes, monthly mean values (post–outlier exclusion) were selected as the aggregation metric for the interrupted time series analysis to minimize estimator variance associated with medians in small samples.

**Supplementary Figure S2. Robustness and sensitivity analyses of the interrupted time series estimates for DIDO time.** Estimated intervention effects on DIDO time across alternative interrupted time series model specifications. Points denote the estimated immediate intervention effect (level change) and error bars indicate 95% confidence intervals (CIs). Models are grouped as main analysis (robustness; blue) and sensitivity analyses (outlier handling; red; and time-trend specifications; brown). Robustness analyses used alternative aggregation metrics (weighted monthly mean and monthly median), both yielding a consistent direction of effect toward reduced DIDO time. Sensitivity analyses assessed the stability of the estimates under varying outlier-exclusion thresholds and alternative time-trend assumptions, including quadratic terms, interaction terms, and seasonal adjustment. Across model families, the estimated effects remained directionally consistent and of similar magnitude, supporting the robustness of the primary findings. Significance levels are denoted as *** P < 0.001, ** P < 0.01, * P < 0.05; ns, not significant. CI = confidence interval, DIDO = door-in–door-out, ITS = interrupted time series, ns = not significant.

**Supplementary Figure S3. Detailed spectral characteristics and variance decomposition of the DIDO time series. (A) Comparison of Major Cycle Components. (B) Decomposition of Variance Sources.** (A) Comparison of dominant periodic components of the DIDO time series before and after the intervention, showing the amplitude of major cycles and their explained variance. The primary cycle length shifted from 21.3 months pre-intervention to 32.0 months post-intervention, indicating a structural alteration in temporal dynamics. (B) Variance decomposition quantifying the proportion of total variance explained by the dominant periodic component; the variance proportion attributable to the primary cycle decreased from 84.0% before the intervention to 80.5% after the intervention, suggesting attenuation of cyclical dominance and increased stability of the underlying signal. DIDO = door-in–door-out.

**Supplementary Figure S4. Stability and specificity analyses of the mechanistic association between DIDO and downstream time metrics. (A) Intervention-Stratified between DIDO and FMC2W. (B) Intervention-Stratified between DIDO and D2D. (C) Overall Relationship between DIDO and FMC2W. (D) Intervention-Stratified between DIDO and FMC2W. (E) Overall Relationship between D2W and FMC2W. (F) Intervention-Stratified between D2W and FMC2W.** (A) Scatter plots of the association between DIDO time and FMC2W, stratified by pre- and post-intervention periods; comparable slopes across periods indicate stability of the mechanistic relationship linking reduced DIDO to shorter FMC2W, used as a system-level proxy for total ischemic time. (B) Intervention-stratified analysis of DIDO versus D2D, providing supplementary context for the negative control result in Figure 2C. (C–F) Negative control analyses evaluating associations between non-targeted intervals (D2D and D2W) and FMC2W, presented as overall and intervention-stratified scatter plots. The absence of a meaningful relationship supports the specificity of the intervention effect and suggests that downstream in-hospital workflows at PCI-capable centers remained largely unchanged. D2D = door-out–to–door, D2W = door-to-wire crossing time, DIDO = door-in–door-out, FMC2W = first medical contact–to–wire crossing, PCI = percutaneous coronary intervention.

**Supplementary Figure S5. Temporal trends of descriptive resource-utilization indicators. (A) Monthly trend in total stent implantation volume over the study period, with no significant immediate intervention effect (level change: 0.078 stents; P = 0.3021). (B) Monthly trend in mean length of hospital stay, also showing no significant immediate intervention effect (level change: 0.239 days; P = 0.6863). These indicators are presented descriptively. Patient-level cost and utility data were unavailable, and no formal cost-effectiveness analysis was performed.**

**Supplementary Figure S6. Diagnostic assessment of the primary interrupted time series model for DIDO time.(A) Residuals versus fitted values for the primary ordinary least-squares segmented regression model of monthly mean DIDO time. (B) Q-Q plot of standardized residuals. (C) Autocorrelation function (ACF) plot of model residuals. (D) Partial autocorrelation function (PACF) plot of model residuals.** These diagnostic plots were used to evaluate residual distribution, model fit, and potential serial correlation in the primary interrupted time series model. Because formal autocorrelation diagnostics suggested evidence of residual serial correlation, Newey-West robust standard errors and generalized least-squares models with an AR(1) correlation structure were additionally used as sensitivity analyses, as reported in Supplementary Table S4. ACF = autocorrelation function; AR(1) = first-order autoregressive; DIDO = door-in–door-out; ITS = interrupted time series; PACF = partial autocorrelation function.

**Supplementary Figure S7. Quarterly trends in in-hospital clinical outcomes.** **(A) Quarterly event rates of composite complications. (B) Quarterly event rates of in-hospital mortality. (C) Quarterly event rates of new-onset in-hospital heart failure.** Event rates were calculated using the final analytical cohort after 95th-percentile DIDO trimming. The vertical dashed line indicates the intervention period beginning in February 2022. Because the intervention occurred within the first quarter of 2022, 2022-Q1 should be interpreted as an intervention-transition quarter. The figure is intended to provide descriptive temporal context for clinical endpoints, particularly low-frequency outcomes such as mortality, and should not be interpreted as definitive evidence of clinical-outcome causality. DIDO = door-in–door-out.

**Supplementary Figure S8. Association between monthly mean DIDO time and monthly mean FMC-to-wire time.** Scatter plot showing the relationship between monthly mean DIDO time and monthly mean FMC-to-wire time after 95th-percentile DIDO trimming, stratified by pre- and post-intervention periods. Monthly mean DIDO was strongly correlated with monthly mean FMC-to-wire time overall (Pearson r = 0.921, 95% CI 0.876-0.950). The association remained positive in both the pre-intervention period (r = 0.957, 95% CI 0.907-0.980) and the post-intervention period (r = 0.755, 95% CI 0.591-0.859). The DIDO-by-period interaction was not statistically significant (P = 0.5015), indicating no clear evidence that the association between DIDO and FMC-to-wire time differed materially before versus after the intervention. CI = confidence interval; DIDO = door-in–door-out; FMC = first medical contact.
